# Safety Profile of Monoclonal Antibody Compared With Traditional Anticancer Drugs: An Analysis of Henan Province Spontaneous Reporting System Database

**DOI:** 10.3389/fphar.2021.760013

**Published:** 2022-01-25

**Authors:** Zhiming Jiao, Ganyi Wang, Zhanchun Feng, Ziqi Yan, Jinwen Zhang, Gang Li, Qianyu Wang, Da Feng

**Affiliations:** ^1^ School of Medicine and Health Management, Tongji Medical College, Huazhong University of Science and Technology, Wuhan, China; ^2^ College of Public Administration, Huazhong University of Science and Technology, Wuhan, China; ^3^ Medical Products Administration and Center for Adverse Drug Reaction (ADR) Monitoring of Henan, Zhengzhou, China; ^4^ Department of Pharmacy, Tongji Hospital, Tongji Medical College, Huazhong University of Science and Technology, Wuhan, China; ^5^ School of Pharmacy, Tongji Medical College, Huazhong University of Science and Technology, Wuhan, China

**Keywords:** monoclonal antibody, spontaneous reporting system, adverse drug reaction, pharmacovigilance, oncology

## Abstract

**Introduction:** Monoclonal antibody (mAb) is an important treatment option for cancer patients and has received widespread attention in recent years. In this context, a comparative safety evaluation of mAbs and traditional anticancer drugs in real-world is warranted.

**Methods:** ADR reports submitted to Henan Adverse Drug Reaction Monitoring Center from 2016 to 2020 for individuals taking antineoplastic drugs were included. Data were analyzed with respect to demographic characteristics, disease types, polypharmacy, past history of ADRs, system organ class, name of suspected drugs per ADR report, severity, result, impact on the primary disease, and biosimilars.

**Results:** A total of 15,910 ADR reports related to antineoplastic drugs were collected, 575 (3.61%) cases were related to mAbs. Female had more reports of ADRs than male. The ADRs of non-mAbs mainly occurred in 1–3 days after injection (4,929, 32.15%), whereas those of mAbs mainly occurred on the same day (297, 51.65%). Serious ADRs accounted for 30.26% (*n* = 174) of mAb-related reports and 34.46% (*n* = 5,285; four death cases) of non-mAb-related reports, respectively. A total of 495 (86.08%) reports were related to the branded drugs of mAbs. In general, our findings indicate that the female, the population aged 60–79 years, people with a single disease, people who have no ADRs in the past and people who have received treatment regimens were less likely to be affected by the primary disease after receiving mAbs therapy. The signal mining method produced 14 signals, only Sintilimab-Hepatic failure was off-label ADR.

**Conclusion:** This study partly confirmed the safety profile of mAbs. It is unlikely to affect groups such as the female, the population aged 60-79 years, people with a single disease, people who have no ADRs in the past and people who have received treatment regimens. Combined drugs have little effect on the primary disease. By conducting signal mining method, 14 signals were produced, and only one of them was off-label ADR.

## Introduction

As a leading cause of death, cancer has caused approximately 10 million deaths worldwide in 2020 (Bray et al., 2021). Among these, 30% (3 million) of death cases happened in China (Sung et al., 2021). Cancer incidence is on the rise worldwide, and the safety of anticancer drugs has become a major concern (Lazarou et al., 1998). When good drugs go bad, the patients have to face problems due to adverse drug reactions (ADRs) (Giacomini et al., 2007), which are harmful or unpleasant reactions resulting from an intervention related to the use of medicinal products (Edwards and Aronson, 2000). The US Food and Drug Administration (FDA) Adverse Event Reporting System (FAERS) was founded in 1969 to support the FDA’s post-marketing safety surveillance program for drug and therapeutic biologic products. In addition, the European Drug Administration (EMA) created the Eudra Vigilance (EV) in 2001 to monitor ADR events. Though the government of China has established the National Adverse Drug Reactions Monitoring Center in 1989 to monitor the safety of drugs, the National Medical Products Administration (NMPA) did not report the ADR of anticancer drugs until 2014 (National Medical Products Administration, 2015). As a result, there are few studies about ADRs of anticancer drugs in China.

Oncology represents a field of medicine involving the high frontiers of new drugs.With the development of bioinformatics technology in cancer treatment, the mAbs provide a better choice for patients. Extensive studies have shown that the mAbs could improve the overall survival of cancer patients (Miller et al., 2007; Tewari et al., 2014; Tewari et al., 2017; Ren T et al., 2021), and mAbs have lower toxicity and better tolerance (Hamilton et al., 2008; Wolska-Washer and Robak, 2019). However, during treatment with mAbs, patients were threatened with ADRs, such as infusion reactions and rash (Hansel et al., 2010), which are frequently happened clinical symptoms. What’s more, most of patients have to suffer from economic burden for the drug price (Norum et al., 2005). Traditional anticancer drugs still occupy the market due to their affordable price (Wysowski and Swartz, 2005; Piccinni et al., 2011; Goss et al., 2014). However, owing to the limitation of drugs, such as toxicity and mechanism of action, it is difficult to avoid the ADRs of traditional anticancer drugs (Barnes et al., 2017). A literature review by Hartmann (Hartmann and Lipp, 2003) focused toxicity of platinum compounds showed the most prominent adverse effects were gastrointestinal toxicity and myelosuppression. In an investigation of non-cardiac drug-induced heart failure, Slordal (Slørdal and Spigset, 2006) found anthracyclines anticancer drugs may cause cardiomyopathy. These studies provide important insights into the safety of mAbs and traditional anticancer drugs respectively. However, recent studies tend to focus on clinical trials, it is necessary to conduct researches on cancer patients based on real-world data.

A great deal of previous research, which was based on the real-world spontaneous reporting system database, has focused on biosimilars or just a few kinds of mAbs. Kalaivani (Kalaivani et al., 2015) analyzed ADR related to mAbs by using the spontaneous reporting system Vigiflow in India and highlighted the need to strengthen the supervision of mAbs and biosimilars. In traceability, Cutroneo (Cutroneo et al., 2014) considered that traceability of biologicals based on the Italian spontaneous reporting system data is currently limited. To figure out the disparities of ADRs between clinical trials and the real world, Gonzalez (González et al., 2008) selected reports from the Spanish Pharmacovigilance System associated with rituximab and trastuzumab. The results were consistent with the safety profile observed in large randomized clinical trials. Although some studies have been carried out on mAbs based on the spontaneous reporting system database, there are gaps in research for identifying the populations that would benefit from mAbs.

This study aims to compare the safety profiles between mAbs and traditional anticancer drugs based on the real-world data of post-marketing drug assessment. And identify the protected factors or potential risks for populations who use mAbs and traditional anticancer drugs, respectively.

## Methods

### Study Design and Setting

We carried out a cross-sectional study of cancer patients with suspected ADRs based on the Henan Provincial Adverse Drug Reaction Monitoring Center, China. We designed to analyze different variables in the reports—mainly the difference between serious ADRs and normal ADRs.

### Participants

The following inclusion criteria were used: 1) Reported between 2016 and 2020; 2) Reports with certain, probable, and possible relationships of drugs; 3) drugs suspected associated with ADR was antineoplastic drug.

The exclusion criteria were as follows: 1) Reports before 2016 and after 2020; 2) Duplicate records; 3) Missing critical information such as age, drug name, and specific records of ADR; 4) Unreasonable records such as records older than 120 years, the record does not match the age, and negative number pertaining to the occurrence time of ADRs.

### Variables

The demographic characteristics, disease types, polypharmacy, past history of ADRs, system organ class, severity, result, impact on the primary disease, and biosimilars were collected. The ADRs and clinical manifestations were organized according to the Medical Dictionary for Regulatory Activities (MedDRA) (version 24.0). ADR reports with antineoplastic drug were identified from the 2nd level of the Anatomical Therapeutic Chemical (ATC) Classification System (L01-antineoplastic agents). The generic names of drugs were standardized and coded according to the catalog of generic names for common prescription drugs. The catalog was issued by the Ministry of Health of China in 2007. The definition for polypharmacy included the use of five or more medications. The severity of ADR was classified by the reporters and included in the database. Based on the Reporting and Monitoring Administration Measure on ADR issued by the Ministry of health of China (Ministry of Health of The People’s Republic of China, 2011), the “Serious ADRs” (SADRs) was defined as and the other cases were regarded as “Normal ADRs”: 1) Results in death; 2) Is life-threatening; 3) Carcinogenesis, teratogenesis and congenital disabilities; 4) Results in persistent or significant disability/incapacity; 5) Require inpatient hospitalization or prolongation of existing hospitalization; 6) Leading to other important medical events, such as the situations listed above may occur without treatment. In the effect of ADR on the primary disease, we defined patients with “prolong,” “worse,” “left with sequelae,” and “death” as “effect,” and patients with other conditions as “no effect.”

### Data Sources

We classified and analyzed the Henan Provincial Adverse Drug Reaction Monitoring Center data from 2016 to 2020. The center is subordinate to the National Center for ADR Monitoring, China. These data were reported by Henan medical institutions, enterprises, and the public. Because the data generated from the spontaneous report system (SRS), we cannot get ADR incidence rates as the true extent of drug use was unknown, so all the data in the study were frequency of reports.

### Study Size

A total of 394,037 initial data were obtained. According to the inclusion and exclusion criteria, 15,910 records were retained. To prevent the repetitive analysis of some reports, we selected one of the main adverse reactions included.

### Statistical Methods

The demographic characteristics, disease types, polypharmacy, past history of ADRs, system organ class, severity, result, impact on the primary disease, and biosimilars in the report were subjected to descriptive analysis, Fisher exact test and Chi-square test. For the death cases, relevant information was detailed. The binary logistic regression was used to analyze the factors affecting the prognosis of SADRs taking different types of drugs. The variables age, sex, disease types, polypharmacy, and past history of ADRs were set as independent variables. The severity and impact on the primary disease were set as the outcome variables, respectively. To further identify the protective factors to impact the primary disease when using mAb, Mantel-haenszel hierarchical analysis was conducted across sub-characteristics. Odds ratios (ORs) and their 95% confidence intervals (CIs) were used in quantifying the associations between variables and types of drugs. All data analyses were performed using SPSS 24.0 (IBM Corp. Armonk, NY). A *p*-value of less than 0.05 was considered statistically significant.

The amount of each ADR of each drug was sorted for ADR signal mining, which quantifies the qualitative nature of the relationship between drugs and ADRs. In ADR signal mining, the reporting odds ratio (ROR), proportional reporting ratio (PRR), and comprehensive standard method (MHRA) were adopted as measures of disproportionality, which are generally used in detecting the imbalance of target events compared with other events in the database (Evans et al., 2001; van Puijenbroek et al., 2002). When the target drug event combination (DEC) frequency was significantly higher and reached the threshold than the background frequency, a signal was considered generated. The strength of the association between drugs and ADRs was expressed as the ROR and PRR with 95% confidence intervals (CIs). We listed the equations and criteria for the three algorithms in Table 1.

**TABLE 1:** Formulas and criteria for generating signals of ROR, PRR and MHRA.

Method	Formula	Criteria and threshold
ROR	$\text{ROR}=\frac{\left(a/c\right)}{\left(b/d\right)}$	a ≥ 3 and lower limit of 95%CI > 1
	$95\text{\%CI}={\text{e}}^{\text{ln}\left(\text{ROR}\right)\pm 1.96\sqrt{1/\text{a}+1/\text{b}+1/\text{c}+1/\text{d}}}$	
PRR	$\text{PRR}=\frac{a/\left(\mathrm{a+b}\right)}{c/\left(c/d\right)}$	a ≥ 3, PRR ≥ 2 and lower limit of 95%CI > 1
	$95\text{\%CI}=e^{\text{ln}\left(\text{PRR}\right)\pm 1.96\sqrt{\sqrt{1/\text{a}+1/\text{b}+1/\text{c}+1/\text{d}}}}$	
MHRA	${\text{x}}^{2}=\frac{n{\left(|\mathrm{ad}-\mathrm{bc}|-\text{n}/2\right)}^{2}}{\left(\mathrm{a+b}\right)\left(\mathrm{a+c}\right)\left(\mathrm{b+c}\right)\left(\mathrm{c+d}\right)}$	a ≥ 3 and χ2 ≥ 4

a: number of reports containing both the suspect drug and the suspect ADR.

b: number of reports containing the suspect ADR with other medications (except the drug of a).

c: number of reports containing the suspect drug with other ADRs (except the event of a).

d: number of reports containing other medications and other ADRs.

## Results

### Demographic Characteristics

A total of 15,910 ADR reports related to anticancer drugs were collected in Henan Provincial Adverse Drug Reaction Monitoring Center from 2016 to 2020, of which 575 (0.14%) were related to mAbs and 15,335 (3.84%) were reported for traditional anticancer drugs. Except 13 cases in which the sex was unknown, women (8,691) tended to have more ADRs than men (7,206), and the male–female ratio was 1:1.43 with a large discrepancy. Sex difference in drug types was not significant (χ^2^ = 1.355; *p* = 0.244). Except 44 cases in which the age was unknown, the average age of patients with mAb-related ADR was 57.9 years old, which was not significantly different with non-mAb group (*t* = 0.544, *p* = 0.841). Two-thirds of ADR reports for mAbs were collected in 2020 (see Table 2). Moreover, 88.35% of the patients suffered from only a single disease, 93.57% did not use the polypharmacy regimen, and 95.48% reported ADRs for the first time.

**TABLE 2:** Description of ADR reports of mAbs vs. non-mAbs.

Characteristic	mAbs *N* = 575 (%)	Non-mAb *N* = 15,335 (%)	*p* value^a^
Mean age ± SD Age groups (years)	57.94 ± 13.72	57.61 ± 14.28	0.841^b^
0–5	3 (0.52)	193 (1.26)	<0.001
6–17	4 (0.70)	231 (1.51)	
18–34	26 (4.52)	422 (2.75)	
35–59	265 (46.09)	6,685 (43.59)	
60–79	254 (44.17)	7,452 (48.59)	
≥80	23 (4.00)	308 (2.01)	
Sex			
Female	328 (57.04)	8,363 (54.54)	0.244
Male	247 (42.96)	6,959 (45.38)	
Year			
2016	6 (1.04)	1,398 (9.12)	<0.001
2017	19 (3.30)	1834 (11.96)	
2018	33 (5.74)	2,455 (16.01)	
2019	140 (24.35)	3,865 (25.20)	
2020	377 (65.57)	5,783 (37.71)	
Disease types			
Single	508 (88.35)	13,539 (88.29)	0.965
Multiple	67 (11.65)	1796 (11.71)	
Polypharmacy			
Polypharmacy	37 (6.43)	940 (6.13)	0.765
Non-polypharmacy	538 (93.57)	14,395 (93.87)	
Past history of ADRs			
No	549 (95.48)	14,279 (93.11)	0.027
Yes	26 (4.52)	1,056 (6.89)	

a Chi-squared test.

b T test.

### Occurrence Time of ADRs

**FIGURE 1:**
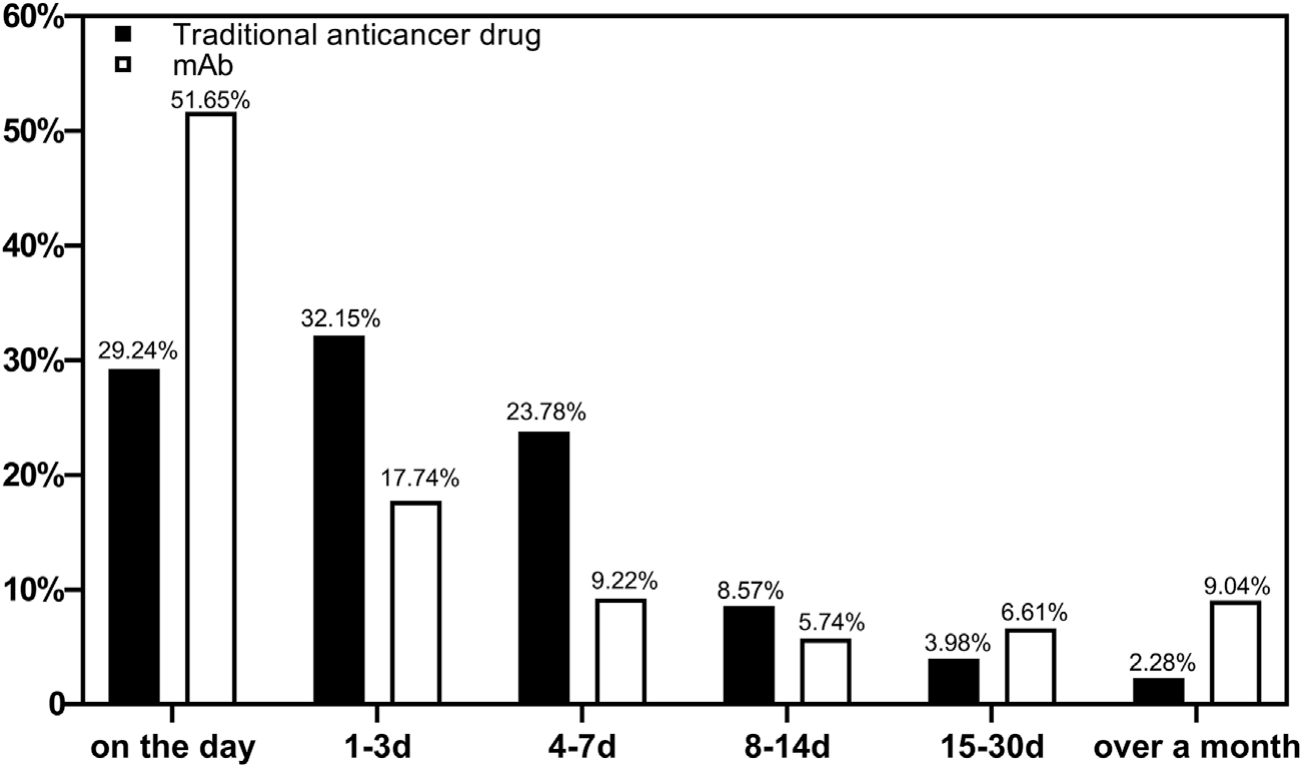
Proportion of reports based on the occurrence time of ADRs by drugs (*n* = 15,904).

The occurrence of ADRs during medication was counted. A total of 15,904 reports were included. Figure 1 shows the occurrence time of ADRs after medications using different types of drugs were started. The ADRs of non-mAbs mainly occurred 1–3 days after use (4,929, 32.15%), whereas those of mAbs mainly occurred on the same day (297, 51.65%).

### System Organ Class of ADRs

A total of 15,910 reports involved a total of 22 system organ class, mainly including gastrointestinal disorders, blood and lymphatic system disorders and skin and subcutaneous tissue disorders. The detailed number and proportion of reports were shown in Table 3.

**TABLE 3:** Number and percentage of ADRs related to system organ damage.

System organ class	mAbs *N* = 575 (%)	Non-mAb *N* = 15,335 (%)	*p* value^a^
Gastrointestinal disorders	81 (14.09)	5,762 (37.57)	<0.001
Blood and lymphatic system disorders	50 (8.70)	3,559 (23.21)	<0.001
Skin and subcutaneous tissue disorders	87 (15.13)	1,263 (8.24)	<0.001
Investigations	20 (3.48)	1,158 (7.55)	<0.001
General disorders and administration site conditions	142 (24.70)	833 (5.43)	<0.001
Nervous system disorders	18 (3.13)	616 (4.02)	0.286
Respiratory, thoracic, and mediastinal disorders	50 (8.70)	578 (3.77)	<0.001
Vascular disorders	22 (3.83)	264 (1.72)	<0.001
Musculoskeletal and connective tissue disorders	5 (0.87)	276 (1.80)	0.096^b^
Cardiac disorders	28 (4.87)	249 (1.62)	<0.001
Hepatobiliary disorders	12 (2.09)	251 (1.64)	0.406
Metabolism and nutrition disorders	5 (0.87)	204 (1.33)	0.341^b^
Immune system disorders	14 (2.43)	166 (1.08)	0.003
Renal and urinary disorders	8 (1.39)	72 (0.47)	0.002
Endocrine disorders	19 (3.30)	17 (0.11)	<0.001
Psychiatric disorders	0 (0)	20 (0.13)	NE
Eye disorders	0 (0)	16 (0.10)	NE
Injury, poisoning, and procedural complications	3 (0.52)	10 (0.07)	0.010^b^
Neoplasms benign, malignant, and unspecified (including cysts and polyps)	11 (1.91)	1 (0.01)	<0.001^b^
Ear and labyrinth disorders	0 (0)	10 (0.07)	NE
Infections and infestations	0 (0)	6 (0.04)	NE
Reproductive system and breast disorders	0 (0)	4 (0.03)	NE

a Chi-squared test.

b Fisher exact test.

NE, not evaluated.

### Severity of the Reported ADRs

Table 4 shows the severity and effects of ADRs. Serious ADRs accounted for 34.31% (*n* = 5,459) of mAb-related reports and 34.46% (*n* = 5,285; four death cases) of non-mAb-related reports. About 83% of the ADRs do not affect the primary disease. The effects of different drug types on the primary disease are significantly different.

**TABLE 4:** Number and proportion of reports on severity and effect of ADRs.

Result	Total N (%)	mAbs No. (%)	Non-mAb No. (%)	*p* value^a^
Total	15,910 (100)	575 (3.61)	15,335 (96.39)	
Severity				
Serious	5,459 (34.31)	174 (30.26)	5,285 (34.46)	0.037
Non-serious	10,451 (65.69)	401 (69.74)	10,050 (65.54)	
Impact on the primary disease				
No effect	13,260 (83.34)	506 (88.00)	12,754 (83.17)	<0.01^b^
Prolong	133 (0.84)	10 (1.74)	123 (0.80)	
Worse	2,499 (15.71)	58 (10.09)	2,441 (15.92)	
Left with sequelae	14 (0.09)	1 (0.17)	13 (0.08)	
Death	4 (0.03)	0 (0)	4 (0.03)	

a Chi-squared test.

b Fisher exact test.

Of the four dead cases, three were over 60 years old, and two were female. The patients mainly suffered from pulmonary, hepatic, and renal diseases. The main ADRs were anaphylactoid reactions, pulmonary fibrosis, hypertension, and pneumonia (see Table 5).

**TABLE 5:** Detailed information of the 4 deaths.

case	Sex	Age	Suspected drug	Time^a^ (day)	Diseases	Dosage	ADR
1	Male	60	RHIL-2	2	Kidney malignant tumor	3 million U	Anaphylactoid reaction
2	Male	73	Gefitinib	58	Respiratory failure; lung carcinoma	250 mg	Pulmonary fibrosis
					Coronary atherosclerosis; diabetes; hypoproteinemia		
3	Female	48	Regorafenib	19	Colonic adenocarcinoma; hepatic metastasis	160 mg	Hypertension; high aminotransferas
4	Female	69	Gefitinib	10	Esophageal carcinoma; lung carcinoma	250 mg	Pneumonia

a The occurrence time of the ADRs.

### Identify Groups that are Relatively Protected From mAbs

The result showed that patients using mAbs (vs. non-mAb, β: 0.418), polypharmacy medication (β: 1.166), and female (vs. male, β: 0.158) were significantly associated with less likely to impact of primary disease (Table 6). In contrast, patients with multiple diseases (vs. single disease, β:0.239), and past ADR history (β:0.587) were observably related with more likely impact primary disease.

**TABLE 6:** Impact and severity by demographic variables and drug types.

Variables	Impact of primary disease		Severity	
	B	OR (95%CI)	B	OR (95%CI)
mAbs (refer to non-mAb)	−0.418^*^	0.658(0.509–0.851)	−0.213^*^	0.808(0.673–0.97)
Age (refer to 18–35)				
0–6	0.361	1.435 (0.941–2.189)	−0.34	0.712 (0.492–1.031)
6–18	0.251	1.286 (0.858–1.927)	0.063	1.065 (0.764–1.483)
35–60	−0.056	0.946 (0.732–1.223)	−0.065	0.937 (0.767–1.146)
60–80	−0.032	0.969 (0.75–1.252)	−0.086	0.918 (0.751–1.122)
>80	0.118	1.125 (0.77–1.644)	−0.033	0.967 (0.715–1.307)
Female (refer to male)	−0.158**	0.854(0.783–0.932)	−0.237**	0.789(0.737–0.845)
With multiple diseases (refer to single disease)	0.239^*^	1.27(1.102–1.463)	0.175^*^	1.191(1.071–1.325)
Polypharmacy	−1.166**	0.312(0.268–0.362)	−0.859**	0.423(0.369–0.486)
Have past ADR history	0.587**	1.799(1.488–2.176)	0.587**	1.799(1.553–2.084)

OR, odds ratio; CI, confidence interval; ^*^ *p* < 0.05, ^**^ *p* < 0.0; NE, not evaluated.

In the issue of severity of ADR, patients using mAbs (vs. non-mAb, β: 0.213), female (vs. male, β: 0.237), and polypharmacy medication (β: 0.859) were significantly associated with non-serious ADRs. On the contrary, patients with multiple diseases (vs. single disease, β:0.175), and past ADR history (β:0.587) were more likely to suffer from serious ADR.

**FIGURE 2:**
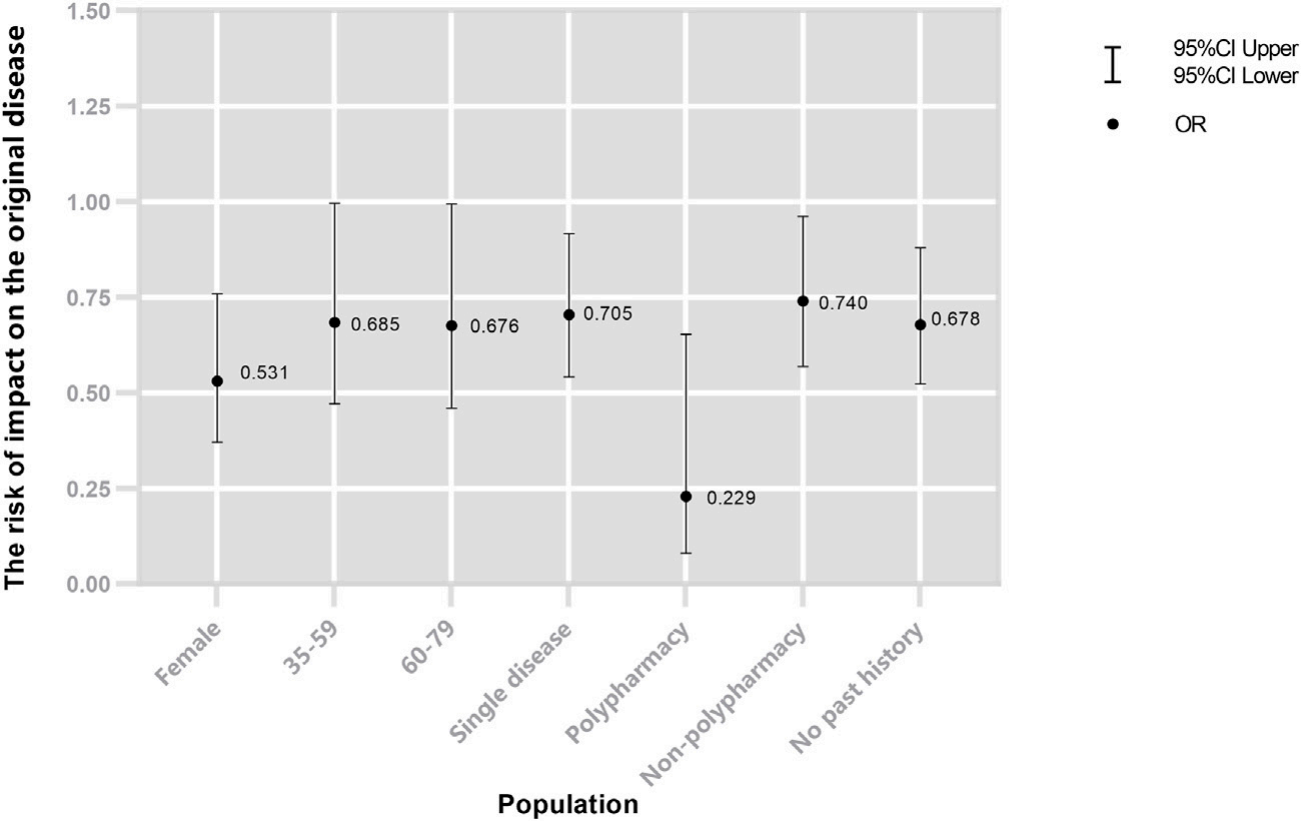
Risk of impact on the primary disease of mAbs.

The results of the stratified analysis showed that female (OR = 0.531, 95% CI: 0.371–0.760), age group of 35–59 years (OR = 0.685, 95% CI: 0.471–0.997), 60–80 years (OR = 0.676, 95% CI: 0.459–0.994), with single disease (OR = 0.705, 95% CI: 0.541–0.917), and no past ADR history (OR = 0.678, 95% CI: 0.524–0.880) were less likely to impact the primary disease after mAbs treatment. Although mAbs were a protective factor in people receiving all treatment regimens, the polypharmacy had less effect on the primary disease (see Figure 2).

### ADR Related to Different Types of Monoclonal Antibodies

There are four classifications of mAbs: murine, chimeric, humanized, and fully human (Buss et al., 2012). The reports showed that no murine mAbs was used. Table 7 shows the most frequent ADRs of different types of mAb. Rash, fever, and chills are common ADRs cross all types of mAbs. Myelosuppression did not occur in the fully human antibodies group.

**TABLE 7:** Most frequent ADRs of three types of mAb.

Types of mAbs (n,%)	Most frequent ADRs of this type (top 5)	No.(%)
Chimeric antibodies		
Rituximab(130,22.61)	Rash	35 (20.83)
Cetuximab(38,6.61)	Chills	22 (13.10)
	Fever	18 (10.71)
	Dyspnea	17 (10.12)
	Myelosuppression	12 (7.14)
Humanized antibodies		
Trastuzumab(171, 29.74)	Fever	44(12.46)
Bevacizumab(77,13.39)	Chills	39(11.05)
Camrelizumab(60,10.43)	Myelosuppression	33(9.35)
Toripalimab(15,2.61)	Vomiting	23(6.52)
Pembrolizumab(10,1.74)	Nausea	22(6.23)
Pertuzumab(9,1.57)		
Nimotuzumab(7,1.22)		
Tislelizumab(2,0.35)		
Atezolizumab(1,0.17)		
Tocilizumab(1,0.17)		
Fully human antibodies		
Sintilimab(48,8.35)	Rash	10(18.52)
Evolocumab(3,0.52)	Fever	5(9.26)
Nivolumab(2,0.35)	Nausea	4(7.41)
Adalimumab(1,0.17)	Hyperthyroidism	4(7.41)
	Hepatic failure	3(5.56)

### Biosimilars

Of the 575 reports of mAbs drugs patents, 495 (86.08%) were related to the branded drugs, 80 (13.91%) reports were concerned with biosimilars. Table 8 shows the difference between the two groups is significant in class “respiratory, thoracic, and mediastinal disorders” (*p* = 0.03).

**TABLE 8:** Number and percentage of ADRs related to system organ class by patents.

System organ class	Branded drugs *N* = 495 (%)	Biosimilars *N* = 80 (%)	*p* value^a^
General disorders and administration site conditions	122 (24.65)	20 (25)	0.95
Skin and subcutaneous tissue disorders	75 (15.15)	12 (15)	0.97
Gastrointestinal disorders	74 (14.95)	7 (8.75)	0.14
Blood and lymphatic system disorders	41 (8.28)	9 (11.25)	0.38
Respiratory, thoracic, and mediastinal disorders	38 (7.68)	12 (15)	0.03
Cardiac disorders	23 (4.65)	5 (6.25)	0.35^b^
Vascular disorders	19 (3.84)	3 (3.75)	0.63^b^
Investigations	17 (3.43)	3 (3.75)	0.54^b^
Endocrine disorders	18 (3.64)	1 (1.25)	0.23^b^
Nervous system disorders	15 (3.03)	3 (3.75)	0.47^b^
Immune system disorders	12 (2.42)	2 (2.5)	0.60^b^
Hepatobiliary disorders	11 (2.22)	1 (1.25)	0.49^b^
Neoplasms benign, malignant, and unspecified (including cysts and polyps)	11 (2.22)	0 (0)	NE
Renal and urinary disorders	6 (1.21)	2 (2.5)	0.31^b^
Metabolism and nutrition disorders	5 (1.01)	0 (0)	NE
Musculoskeletal and connective tissue disorders	5 (1.01)	0 (0)	NE
Injury, poisoning, and procedural complications	3 (0.61)	0 (0)	NE

a Chi-squared test.

b Fisher exact test.

NE, not evaluated.

### Signal Mining

According to the calculation formulas and thresholds, DEC signals that do not meet the criteria were excluded. The three signal mining methods produced a total of 14 signals (see Table 9). The strength of the correlation between the drug and ADR increased with the ROR and PRR values.

**TABLE 9:** Signals of ADRs.

Drug	ADR	ROR	95% CI lower limit	PRR	95% CI lower limit	χ^2^
Pembrolizumab	Pneumonitis	39.93	8.28	28.25	8.77	104632.48
Rituximab	Urticaria	10.49	1.08	10.27	1.09	16193.88
Sintilimab	Hepatic failure^a^	6.96	1.61	6.59	1.62	9109.80
Sintilimab	Hyperthyroidism	9.49	2.46	8.78	2.48	6804.49
Camrelizumab	Hyperthyroidism	7.74	2.02	7.27	2.03	5081.48
Camrelizumab	Pneumonitis	4.74	1.15	4.54	1.16	5049.10
Camrelizumab	Hypothyroidism	9.87	2.76	9.09	2.79	3780.48
Bevacizumab	Hypertension	12.35	3.53	11.32	3.55	1691.69
Nimotuzumab	Fever	5.91	1.29	3.80	1.50	457.11
Bevacizumab	Vomiting	3.28	1.36	3.04	1.38	197.76
Trastuzumab	Vomiting	2.27	1.01	2.18	1.03	73.28
Sintilimab	Rash	2.63	1.23	2.29	1.26	42.36
Rituximab	Dyspnea	5.15	2.30	4.67	2.33	22.22
Trastuzumab	Chills	3.74	2.17	3.18	2.22	5.98

a Off-label ADR.

## Discussion

This study aims to examine the safety profile of mAbs compared with non-mAbs. To our knowledge, this study is the first report in China to explore the safety of mAbs using the data of Henan Provincial Adverse Drug Reaction Monitoring Center. Our study showed that approximately 0.144% of whole reports related to mAbs drugs, and more than two-thirds of mAb-related ADR reports were reported in 2020 in our study. In recent years, the catalog of medicines covered by the national medical insurance system has included an increasing number of mAbs in China (National Healthcare Security Administration, 2020). This situation is in line with the increasing number of mAb-related reports. In addition, as the policy of drug centralized procurement (Sunshine Medical Procurement All-In-One, 2021), which takes more than 70 biological varieties, such as rituximab, trastuzumab, and bevacizumab into the procurement list, the number of mAb-related ADR reports is expected to increase in the next few years. This increase means that the authorities have more information about mAbs and more accurate decisions of policy.

The difference between age groups is not significant. This result differs from a study about biological medicines in Italy, in which patients treated with biologicals were elderly people (Cutroneo et al., 2014). The possible reason was the small sample size of mAbs, which does not allow us to draw firm conclusions.

The male to female ratio was 1:1.43, but due to the limitation of the database, the total number of patients using suspected drugs was unknown. The difference in cancer incidence indicates that even if the number of reports differs between males and females, this does not mean that it is a gender difference (Jiao et al., 2021). And no evidence of an association (*p* = 0.244) between gender and ADR severity was found. This finding is similar to Hendriksen’s study (Hendriksen et al., 2021), which showed that women have a 1.5–1.7 times higher risk of severe ADRs than men, but the rate of severe ADRs did not differ between women and men.

Our study showed that 51.65% of the total mAb-related ADRs were found on the day of use. Early observation played a key role in the use of mAbs. Family members should be reminded to monitor them closely on the day of use so that most ADRs can be detected as early as possible and treated in time. Meanwhile, 32.15% of ADRs with non-mAbs occurred 1–3 days after use, and 85.17% of the ADRs occurred within 1 week. Hence, a long period of monitoring and care is required for non-mAb medicated patients to detect long latent ADRs promptly.

The percentage of serious ADRs reports and impact on the primary disease reports are lower for mAbs than for the non-mAb group (30.26 vs. 34.46%; 12 vs. 16.83%). Preclinical experience suggests a better tolerance to the treatment with mAbs than to the non-mAbs (González et al., 2008). Rituximab is associated with a low incidence of severe ADRs, in contrast to non-mAbs (Kimby, 2005). In a study of 62 patients receiving rituximab, ADRs were limited to the typically related infusion reactions. Only two patients (3%) experiencing serious toxicity after the infusion, and no additional serious ADRs were reported (Hainsworth, 2002). MAbs, such as trastuzumab, combined with traditional chemotherapy are superior to the anticancer drugs alone in terms of overall survival, response rate, and tolerance, and have low toxicity and few ADRs (Baselga et al., 2012a; Baselga et al., 2012b; Gianni et al., 2012).

In our analysis, reports on “general disorders and administration site conditions,” “neoplasms benign, malignant, and unspecified (including cysts and polyps),” and “endocrine disorders” were more likely about mAbs. First, nearly all mAbs share a risk of infusion-related reactions (headache, fatigue, nausea, vomiting, fever, and chills) and a high enough frequency to warrant special precautions (Brown and Torabi, 2011; Wei et al., 2015; Bayer, 2019). The frequent ADR reports of mAbs related to general disorders also reflect this result (Guan et al., 2015). Second, all “neoplasms benign, malignant, and unspecified” related to ADRs were hemangioma cause by camrelizumab (Nie et al., 2019; Song et al., 2019). The mechanism of hemangioma occurrence is still unclear but may be attributed to immune stress responses of the cutaneous capillary endothelial cells (Wang et al., 2020). Third, all endocrine disorders related to ADRs were thyroid dysfunction. The possible reason is antibody-mediated thyroid autoimmunity (Coles et al., 1999; Coles et al., 2008), which occurred in almost 25% of subjects in a study of 334 patients (Coles et al., 2008). These ADRs that are more relevant to mAbs mean that physicians should also consider working with nurses to develop procedures for the safe handling of these drugs (Bayer, 2019).

This study provides important information that mAbs appear to have a protective effect on certain subgroups, with less impact on primary disease, and this is particularly evident in certain groups, such as women and the elderly. This finding may be because most of the mAbs are used for the breast cancer of female (trastuzumab, pertuzumab, and bevacizumab), thus women may be more affected. MAbs with better effects and less adverse reaction profile, making it particularly justifiable for old patients (Coiffier et al., 2002; Thomas et al., 2006; Kantarjian et al., 2018). Notably, the polypharmacy group was easier protected by mAbs (OR = 0.23 vs. OR = 0.74). This result is consistent with the mAbs combination. Trastuzumab (de Azambuja et al., 2014; Swain et al., 2020), rituximab (Pfreundschuh et al., 2006; Furman et al., 2014), and bevacizumab (Kato et al., 2018; Finn et al., 2020) proved to work better in combination with traditional anticancer drugs.

We found that patients who had no past ADR history were less likely to impact the primary disease after using mAbs. This result is related to the characters of biologics, with the elements that may be recognized by the recipient as foreign, mAbs would cause the activation of immune reactions (Presta, 2006; Giezen et al., 2008), which commonly occur after initial dosing (Klastersky, 2006; Chung, 2008). Thus, for the patients who have no past ADR history, the risk of continuing to use mAbs may be reduced. However, physicians should still be cautious when prescribing mAbs. This finding was reflected in the reports. 63.65% of physicians abandoned mAbs after occurred ADRs and did not continue using them.

Prior mAbs were generated by animals (most were mice) and were used for therapy. Unfortunately, the administration produced allergic reactions and no clinical benefit in many patients (Modjtahedi et al., 2012). To decrease the severity and frequency of ADRs, new generations of mAbs were employed, such as humanized and fully human mAbs. We found that some common ADRs—such as rash, chills, and fever—occurs in all three mAbs types, but the ADR related to the immune systems, such as myelosuppression, only accounts for a small part of ADR of fully human antibodies. This may because fully human mAbs are less antigenic and better tolerated compared with the other classes of mAbs, which means the fully human mAbs is probably the inevitable choice for the development of antibody drugs.

Concerning biosimilars, a low number of ADR reports were received in the Henan Province, in line with the low penetration of these drugs in the market. In China, biosimilar drug has not been approved until 2019 because of the lack of national biosimilar regulatory guidance (Xu et al., 2019). Moreover, the development costs of biosimilars are significantly higher than their generic equivalents due to therapeutic equivalence trials and higher manufacturing costs (Akram et al., 2021), so the biosimilars have not broken the monopoly of patented drugs yet. However, given that China’s drug development capacity has dramatically improved over the past decade (Wu et al., 2015), as further development of China’s drug reform creates an efficient review and regulatory process (Zhu and Liu, 2020), it is expected that a large number of biosimilars will emerge in the next few years, which may contribute to the sustainability of the healthcare system.

In the context of widespread use of anticancer drugs, some certain ADRs of certain mAbs may generate signals, which means that the ADR and mAbs were probably related. Combined with drug instructions, only sintilimab-hepatic failure was off-label ADR. Hepatic failure seemed to be a rare ADR of sintilimab, and studies on the ADRs of sintilimab are few. Only one case was reported in related studies (Ren Z et al., 2021). However, in view of the description of the reported data and positive signal, further research is needed.

## Limitations

In China, ADR reports were reported by basic units (including drug manufacturers, pharmacies, and medical institutions) to provincial ADR monitoring centers. And then, the reports were evaluated by provincial and national ADR monitoring centers. The strict evaluation process ensures the accuracy of ADR reports, but the high threshold may leads to a higher rate of underreporting. Probably a much higher number of ADRs have occurred in real life. In our research, almost all reports came from hospitals. We encourage consumers and non-medical personnel to report ADRs to the system to better assess ADRs comprehensively and reduce bias, and it is also recommended that more studies conduct more prospective investigations, and further investigate the observed signals based on the database of non-spontaneous reports, so as to better estimate the type and incidence of ADR.

This study has several limitations. First, this study used the database of Henan Province, which does not necessarily represent the actual situation of the whole country. Second, owing to the limitation of the selected signal mining method, the combination of drugs is not considered, and the conclusion may be biased. It must be said that the signal detection method is based on the reported quantitative correlation rather than biological correlation, and cannot represent the inevitable causal relationship between drugs and adverse reactions. The determination of the causal relationship which includes the potential impact of false positives or false negatives needs further evaluation and verification. Third, the sample database analyzed in this study is all recorded ADR data and does not consider the occurrence of ADRs in the same patient in different years, making the analysis results biased.

Due to the limitation of the signal mining method and sample quantity, the results cannot represent the inevitable causal relationship between drugs and adverse reactions. The causal relationship, which includes the potential impact of false positives or negatives, needs further evaluation and verification. The methods we used—including ROR, PRR and MHRA—were frequentist statistical approaches, which means the limits of detecting false-positive signals and low specificity were unavoidable (Noguchi et al., 2021). When the number of reports is small, its stability will be greatly affected. Thus, signal mining aims to detect unknown ADR signals and provide more information and references.

## Conclusion

In general, our findings indicate that the female, the population aged 60–79 years, people with a single disease, people who have no ADRs in the past and people who have a single disease, had no history of adverse reactions and had received treatment regimens were less likely to be affected by the primary disease after receiving mAbs therapy. In signal mining, 14 signals were produced, and only one of them was off-label ADR. Although the safety of mAbs has been confirmed by various studies, physicians should be cautious when prescribing mAbs medications, and patients using mAbs need to be monitored and cared for closely on the day of use.

## Data Availability

The original contributions presented in the study are included in the article/supplementary files, further inquiries can be directed to the corresponding author.
